# The ubiquitin-like protein Hub1/UBL-5 functions in pre-mRNA splicing in *Caenorhabditis elegans*

**DOI:** 10.1002/1873-3468.14555

**Published:** 2022-12-19

**Authors:** Kiran Kumar Kolathur, Pallavi Sharma, Nagesh Y. Kadam, Navneet Shahi, Ane Nishitha, Kavita Babu, Shravan Kumar Mishra

**Affiliations:** 1 Department of Biological Sciences, Indian Institute of Science Education and Research (IISER) Mohali, Punjab, India; 2 Department of Pharmaceutical Biotechnology, Manipal College of Pharmaceutical Sciences (MCOPS), Manipal Academy of Higher Education (MAHE), India; 3 Centre for Neuroscience, Indian Institute of Science, Bangalore, India

**Keywords:** outron-containing transcripts, pre-mRNA splicing, spliceosome, *trans*-splicing and *C. elegans*

## Abstract

The ubiquitin-like protein Hub1/UBL-5 associates with proteins non-covalently. Hub1 promotes alternative splicing and splicing of precursor mRNAs with weak introns in yeast and mammalian cells; however, its splicing function has remained elusive in multicellular organisms. Here, we demonstrate the splicing function of Hub1/UBL-5 in the free-living nematode *Caenorhabditis elegans*. Hub1/UBL-5 binds to the HIND-containing splicing factors Snu66/SART-1 and PRP-38 and associates with other spliceosomal proteins. *C. elegans hub1/ubl-5* mutants die at the Larval 3 stage and show splicing defects for selected targets, similar to the mutants in yeast and mammalian cells. UBL-5 complemented growth and splicing defects in *Schizosaccharomyces pombe hub1* mutants, confirming its functional conservation. Thus, UBL-5 is important for *C. elegans* development and plays a conserved pre-mRNA splicing function.

The majority of eukaryotic genes are interrupted by non-coding introns that are precisely excised out to produce mature RNAs by a process known as RNA splicing. Pre-mRNA exons are either constitutively spliced, becoming part of a single mRNA, or are alternatively spliced, producing mRNA variants [1]. Both splicing processes are carried out by two transesterification reactions by a large ribonucleoprotein complex, the spliceosome [2]. In contrast to humans, where more than 90% of genes are alternatively spliced [3], in *Caenorhabditis elegans*, ~ 35% of genes undergo alternative splicing, but, strikingly, ~ 85% of the genes are *trans*-spliced. In this process, a 22 nucleotides (nt) splice leader (SL) RNA coded by distinct gene splices to the 5′ ends of transcripts coded by a different set of genes. Thus, a pre-mRNA undergoing *trans*-splicing contains a 3′ splice site (SS) but lacks a 5’ SS, which is provided by the SL RNA [SL RNAs resemble small nuclear RNAs (snRNAs)] [4–8]. The SL small nuclear ribonucleoprotein (snRNP) donates the 22-nt SL to the *trans*-splice site (3’ SS on the pre-mRNA) [9].

The *C. elegans* genome contains more than 1000 operons, and the resulting polycistronic RNA is cut into monocistronic units by 3′-end formation and *trans*-splicing. SL1 and SL2 *trans*-splicing occur in different genes. Non-operon genes and the first gene in an operon are *trans*-spliced to SL1. However, SL2 *trans*-splicing is mainly used for splicing polycistronic pre-mRNA [10].

The ubiquitin-like protein Hub1 (also referred to as UBL-5 in *C. elegans*) is a conserved member of the UBL family in eukaryotes reported to function in pre-mRNA splicing [11–17]. In the budding yeast, *Saccharomyces cerevisiae*, Hub1 binds directly, but non-covalently, to Hub1 interaction domain (HIND) of the splicing factor Snu66, a component of the U4/U6.U5 tri-small nuclear ribonucleoprotein (tri-snRNP), and promotes excision of introns with weak 5’SS [13,15,16]. It also interacts with the DEAD-box helicase Prp5, a regulator of pre-spliceosome assembly, and activates its ATPase activity, thereby enhancing overall splicing efficiency. Interestingly, Hub1-mediated splicing through the non-canonical 5’SS in *SRC1* and *PRP5* transcripts also promotes their alternative splicing [15,16,18,19]. Consequently, elevated levels of Hub1 affect spliceosomes’ fidelity and causes aberrant splicing by tolerating suboptimal SSs and branch-point sequence (BPS) [18]. Stress-induced upregulation of Hub1 in *S. cerevisiae* promotes splicing of introns with non-canonical SSs to improve stress tolerance [20].

While Hub1’s splicing function has been well studied in yeast and cultured human cells, a similar role has not been reported in multicellular animals, including the nematode *Caenorhabditis elegans*. In this organism, UBL-5 has been reported to function in mitochondrial unfolded protein response (UPRmt) by associating with the transcription factor DVE-1 for inducing the expression of the mitochondrial chaperones HSP-60 and HSP-6. However, pre-mRNA splicing defects could not be observed in *ubl-5* mutant worms [21,22]. Notably, both UBL-5 and homologues of the HIND-containing splicing factors Snu66 and Prp38 are present in *C. elegans*. Thus, UBL-5 might play a role in RNA splicing. It has been independently suggested that UBL-5’s role in UPRmt could be an outcome of its pre-mRNA splicing function [23].

In this study, we have investigated the potential role of *C. elegans* UBL-5 in pre-mRNA splicing. The *ubl-5* mutant animals did not grow beyond the Larval 3 stage, indicating a crucial role of UBL-5 in the animal’s development. By making a splicing-sensitive microarray of a subset of genes and hybridizing total RNA isolated from L3-stage mutant worms, we found an accumulation of intron-containing transcripts, outron-containing transcripts and intercistronic regions in *ubl-5* mutant worms. The data presented here suggest that UBL-5 plays a role in *cis*- and *trans*-RNA splicing.

## Methods summary

Yeast strains described in this study are listed in Tables S3 and S4.

Protocols used for protein purification, protein–protein interaction, *Schizosaccharomyces pombe* complementation, splicing assays, *C. elegans* strain maintenance and rescue experiments are described in the Data S1 section.

## Results

### *C. elegans* UBL-5 binds to HIND-containing spliceosomal proteins

**Fig. 1:**
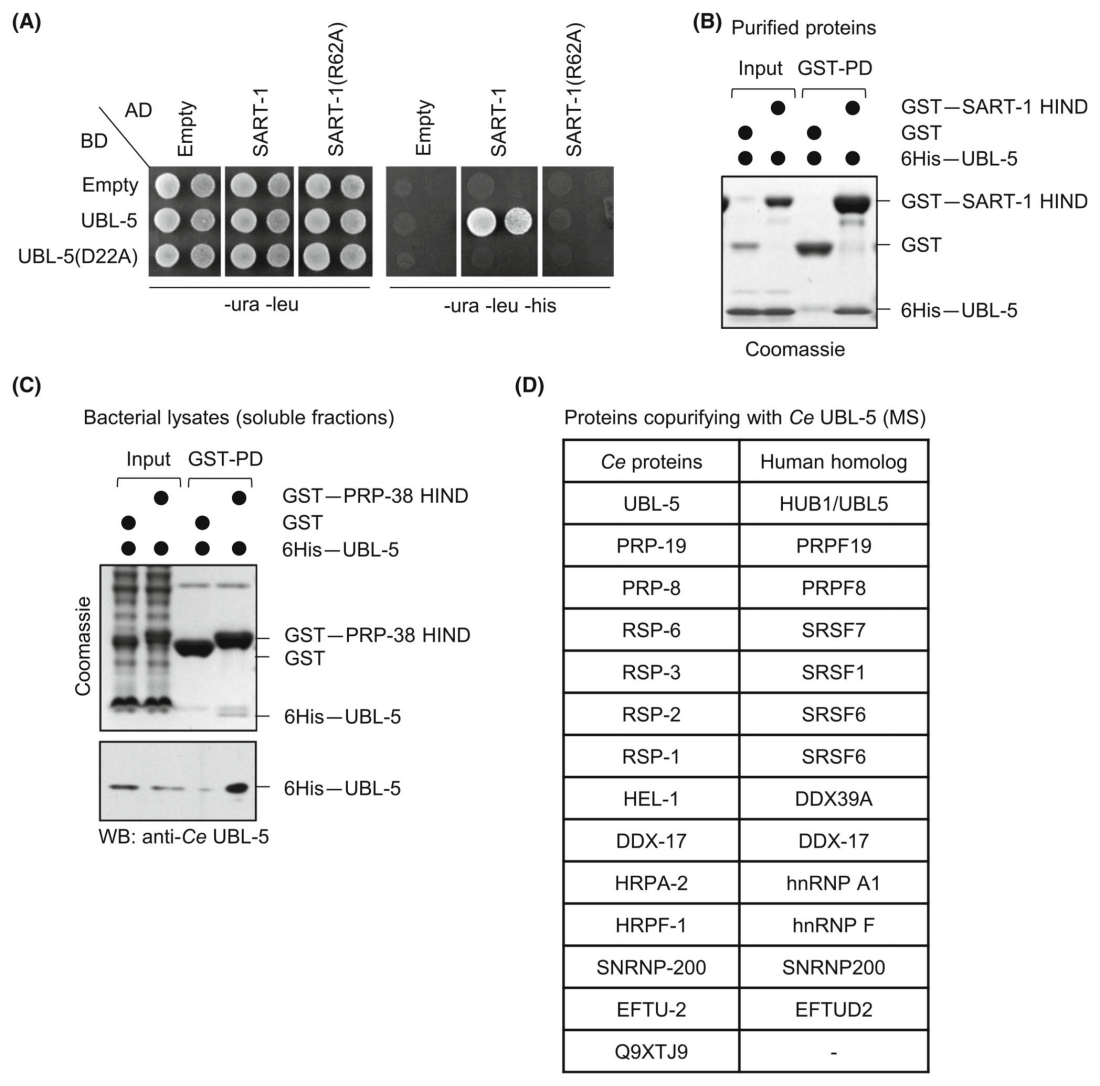
*Caenorhabditis elegans* UBL-5–HIND interaction. (A) Yeast two-hybrid interaction assay (Y2H). Conserved mode of interaction between UBL-5 and SART-1 through salt bridge forming residues in *C. elegans*. Plasmids expressing *C. elegans* UBL-5 and UBL-5(D22A) mutant fused to Gal4-binding domain (BD; with uracil marker) and SART-1 HIND and SART-1 HIND(R62A) mutant fused to activation domain (AD; with leucine marker) were expressed in yeast cells. Transformants were five-fold diluted and spotted on control (-ura -leu) and selective (-ura -leu -his) plates. The expression of the *HIS3* reporter gene, which allows growth on -his selection medium, indicates interaction between the two fusion proteins. The UBL-5(D22A) mutant did not bind SART-1 HIND, and SART-1 HIND(R62A) mutant did not bind UBL-5. (B) *C. elegans* UBL-5 interacts with SART-1 HIND. GST pulldown assays with recombinant GST–SART-1 HIND fusion protein and 6xHis–UBL-5. GST–SART-1 HIND bound UBL-5. GST was used as a negative control. Input represents about one-tenth of the total proteins used in the pulldown. (C) *C. elegans* UBL-5 interacts with PRP-38 HIND. GST pulldown assays from lysates of bacteria (*Escherichia coli*) expressing GST–PRP-38 HIND fusion protein and 6xHis–UBL-5. GST–PRP-38 HIND bound UBL-5. GST was used as a control. Input represents about one-tenth of the total proteins used in the pulldown. GST–PRP-38 HIND pulldown samples were immunoblotted with a *C. elegans* UBL-5 antibody raised in rabbits. (D) UBL-5 co-immunoprecipitates with the splicing factors in worms. 3xFlag–UBL-5-expressing *C. elegans* cells were immunoprecipitated using an anti-FLAG antibody, and bound proteins were analysed by mass spectrometry (MS). The table shows a list of splicing factors co-purified with UBL-5 (MS analysis of anti-FLAG IP material from untagged worms was used as negative control). Human homologues are shown for illustration. *Hs, Homo sapiens*; *Ce, Caenorhabditis elegans*.

Hub1 binds to HIND-containing pre-mRNA splicing factor Snu66 to promote alternative splicing in yeast and human cell lines [14,16]. We observed two putative HIND elements in *C. elegans* proteins; at the N terminus of Snu66/SART-1 and the C terminus of PRP-38 (Fig. S1). To verify whether *C. elegans* UBL-5 binds to the putative elements in these proteins, we carried out yeast two-hybrid assays. Yeast cells transformed with *C. elegans* UBL-5 and SART-1 (HIND) constructs were selected, and transformants were grown on plates lacking histidine. The growth on these plates is indicative of the interaction (Fig. 1A). The importance of the salt bridge between Asp22 on UBL-5 and Arg62 on SART-1 for the interaction was confirmed by altering the residues, which abolished the interaction (Fig. 1A). Thus, we infer that the binding of *C. elegans* UBL-5 to SART-1 is also mediated through a salt bridge, showing a conserved mode of interaction between these proteins in *C. elegans*. However, yeast cells transformed with *C. elegans* UBL-5 and PRP-38 (HIND) constructs, lacked growth on plates lacking histidine (data not shown). The absence of two-hybrid interaction between UBL-5 and PRP-38 may be attributed to one or more of the following reasons; PRP-38 (HIND) harbours a weaker UBL-5-binding motif when compared to SART-1 (HIND), possible transient interaction between PRP-38 and UBL-5, technical limitations of the assay.

To understand UBL-5-HIND interaction further, we performed GST pulldown assays. The recombinant fusion protein of SART-1 (HIND) with GST was able to pulldown UBL-5, which confirmed a direct interaction between them (Fig. 1B). To verify whether *C. elegans* UBL-5 binds to the putative HIND elements of PRP-38, we carried out the GST pulldown assay using bacterial lysates containing soluble proteins. We were unsuccessful in purifying full-length recombinant GST-PRP-38 (HIND)-containing protein and thus used crude bacterial lysate. GST-PRP-38 (HIND)-containing protein lysate was able to pulldown UBL-5 (Fig. 1C). The interaction was verified further by immunoblotting with a UBL-5-specific antibody (Fig. 1C). Thus, *C. elegans* UBL-5 interacts with the HIND-containing splicing factors SART-1 and PRP-38, suggesting a potential role of UBL-5 in pre-mRNA splicing.

To test if UBL-5 interacted with the spliceosome or splicing factors in *C. elegans*, worms expressing 3xFlag–UBL-5 constructs were immunoprecipitated using anti-FLAG beads. The UBL-5 co-immunoprecipitated complex was eluted and analysed by mass spectrometry. We report the identification of different splicing factors enriched in UBL-5-purified complexes (Fig. 1D and Table S1). Certain splicing factors co-purified with UBL-5 suggest its association with spliceosomal factors *in vivo* in *C. elegans*. We also detected additional proteins related to other biological processes co-purifying with UBL-5 (Table S2). However, the splicing factors co-purifying with UBL-5 represent the spliceosome only partially, either because of weak/transient associations or technical challenges associated with spliceosome purification from the worms. Nonetheless, both *in vitro* and *in vivo* evidence indicate that *C. elegans* UBL-5 associates with components of the spliceosome.

### UBL-5 is essential for the development of *C. elegans*

**Fig. 2:**
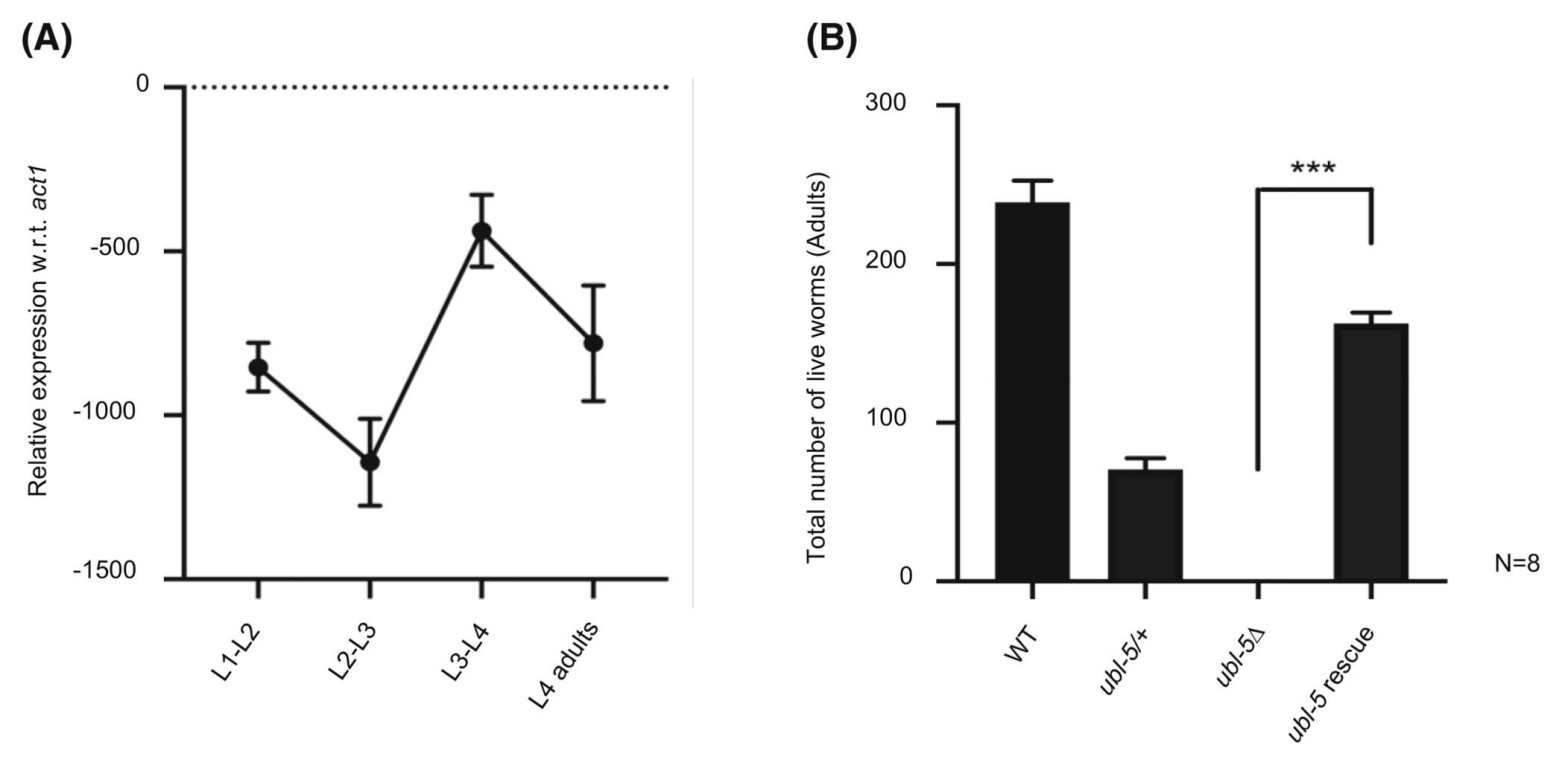
*Ubl-5* expression is critical in *Caenorhabditis elegans*. (A) *ubl-5* expression in the worms. *ubl-5* mRNA was monitored in the L1-L2, L2-L3, L3-L4 and L4-adult stages of the worm using real-time PCR. *ubl-5* levels are normalized against *act-1*. L3-L4 worms showed the highest expression of all other developmental stages. (B) Lethality in adult *ubl-5* knockout worms. A genomic *ubl-5* construct rescued the lethality in *ubl-5* mutants. The total number of adult worms surviving from a single parent worm is counted for WT, *ubl-5*/+, *Δubl-5* and *ubl-5* rescue lines.

UBL-5 is essential for the viability of *C. elegans*. As *ubl-5* mutant worms did not survive after the Larval stage 3 (L3), the mutants were generated by using UV as a mutagen, giving rise to a chromosomal deletion in the *ubl-5* that was balanced using a GFP-marked translocation. Hence, heterozygous worms show pharyngeal GFP signals, while homozygous mutant worms showed early larval arrest [24]. This phenotype suggested that UBL-5 activity is crucial at the L3 stage of worm development. We next carried out an expression profile of the *ubl-5* gene in a larval stage-specific manner in WT animals by quantitative RT-PCR. The expression of *ubl-5* varied in a stage-specific manner; the transcript levels were higher at the L3 stage of development (Fig. 2A). These data indicate that the expression/activity of *ubl-5* at the L3 stage may be required for the normal development of worms. Furthermore, to confirm that the lethality of the mutant worms is due to the absence of *ubl-5*, we expressed a *ubl-5* genomic DNA clone in *ubl-5* mutant worms. The lethality was rescued (Fig. 2B). These results suggested that UBL-5 is essential for *C. elegans* development.

### *C. elegans ubl-5* mutants show defects in *cis*- and *trans*-splicing

**Fig. 3:**
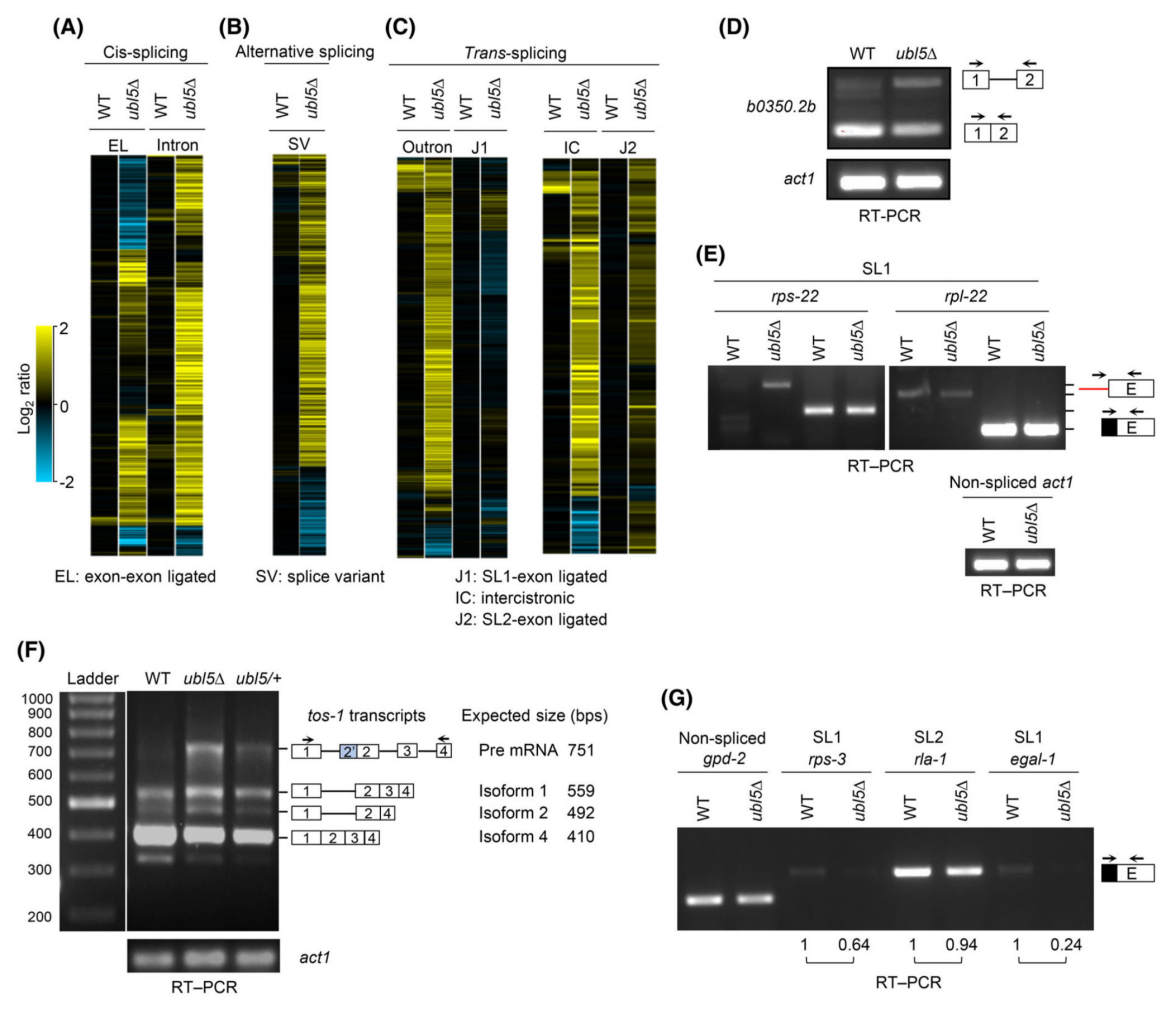
UBL-5 is required for *cis*- and *trans*-splicing in *Caenorhabditis elegans*. (A) Analysis of total RNA from WT and *ubl-5* mutant worms using a splicing-sensitive microarray to monitor *cis*-splicing events. The microarray heat map represents the log2-fold-change values of the mutant samples compared to WT samples for exon–exon ligated (EL) and intron-containing transcripts. Yellow represents accumulation, black denotes no change and blue shows a reduction in signals. From 439 targets analysed for *cis*-splicing using intron-specific probes, 49 targets showed ≥ 2-fold change (*P* ≤ 0.05). ≥ 2-fold changes were chosen as a conservative estimate (stringent criteria). Statistical significance was *P* ≤ 0.05. *q*-value is not used due to a lower sample number. (B) Analysis of alternative splicing events using the splicing-sensitive microarray. The mutant samples are compared to WT samples for mRNA-specific splice variants (SV). From 570 targets analysed for alternative splicing using splice variant probes, 41 targets showed ≥ 2-fold change (*P* ≤ 0.05). (C) Analysis of *trans*-splicing events using splicing-sensitive microarray. The mutant samples are compared to WT samples for outron and SL1 *trans*-spliced transcripts (J1). From 1267 targets analysed using *trans*-splicing outron probes, 94 targets showed ≥ 2-fold change (*P* ≤ 0.05). The mutant samples are compared to WT samples for intercistronic regions containing transcripts (IC) and SL2 *trans*-spliced transcripts (J2). And, from 156 targets analysed using SL2 *trans*-splicing intercistronic probes, 9 targets showed ≥ 2-fold change (*P* ≤ 0.05). (D) *C. elegans* UBL-5 is required for *cis*-splicing. Semi-quantitative RT-PCR reveals the accumulation of intron-containing *b0350.2b* transcripts. The block diagrams (not drawn to the scale) represent exons and introns. Primers are depicted with arrows on exons. RT-PCR of *act1* (actin) pre-mRNA is used as a control. (E) Semi-quantitative RT-PCR for outron-containing transcripts. Primers are indicated with arrows. Forward primers were specific to the SL1 sequence (black box) or the outron (red line). Reverse primers were specific to the exons (unfilled box). RT-PCR of *act-1* (actin) pre-mRNA is used as a control. (F) Semi-quantitative RT-PCR showing accumulation of intron-containing *tos-1* transcripts. The block diagrams (not drawn to the scale) represent exons and introns. 2′ exon is cryptic and provides the 3′ splice site to the preceding intron. Primers are depicted with arrows. RT-PCR of *act-1* (actin) pre-mRNA is used as a control. Lanes unrelated to the probe were cropped to assemble the figure. The unedited image is shown in Fig. S2. PCR product sizes are deduced from [38]. (G) Semi-quantitative RT-PCR showing potential role of *ubl-5* in *trans*-splicing. Forward primers were specific to SL1 and SL2 (black boxes) and reverse primers were specific to the exons (unfilled box). RT-PCR of *gpd-2* pre-mRNA is used as a control. Relative signal intensities in WT and mutant worms are analysed using IMAGEJ software (National Institutes of Health, Bethesda, MA, USA and the Laboratory for Optical and Computational Instrumentation University of Wisconsin, Madison, WI, USA).

To understand the role of UBL-5 in RNA splicing, we designed a splicing-sensitive microarray for a subset of *C. elegans* genes. The array was rich in neuronal genes that undergo alternative splicing or SL1/SL2-mediated *trans*-splicing. It contained probes specific for introns, exon–exon junction and mRNA-specific splice variants (as a measure of alternative splicing) for a subset of genes to monitor *cis*-splicing events (Fig. 3A,B). In addition, various oligonucleotide probes were also included for measuring *trans*-splicing: outronic probes detected pre-mRNA and *trans*-spliced junction probes (SL1) detected mature SL1-mRNA. For SL2 *trans*-splicing, we used 60-bp-long probes, 30 bp upstream and 30 bp downstream of the *trans*-splice site of the candidate gene, for detecting the pre-mRNAs. The *trans*-spliced junction probes (SL2) detected mature SL2-mRNA (Fig. 3C).

While *ubl-5* knockout mutants are lethal in *C. elegans*, the worms survived until the L3 stage. Therefore, the mutant worms were maintained in a heterozygous state with the help of a balancer chromosome. We collected L3-stage WT and *ubl-5* mutant worms, isolated total RNA, reverse transcribed it to double-stranded cDNA, generated labelled cRNA by *in vitro* transcription of the cDNA, and fragmented the cRNA and hybridized on the microarray. The microarray heat maps in Fig. 3A–C represent fold-change values obtained by comparing mutant samples with WT. Splicing patterns of multiple genes were altered in the *ubl-5* mutants, as evidenced by the enhanced accumulation of splice variants, intron-, outron- and intercistronic regions containing transcripts compared to WT worms. We also examined the splicing of *b0350.2b* (selected candidate) and *tos-1* (*tos-1* provides a sensitive readout for studying alternative splicing in *C. elegans*) genes by using RT-PCR experiments [25]. In the case of the *b0350.2b* transcript, an accumulation of intron-containing products was observed in *ubl-5* mutant worms (Fig. 3D). Increased retention of pre-mRNA, skipped 2′ cryptic exon (isoform 1) and skipped exon 3 (isoform 2) of the *tos-1* transcript were also observed in the *ubl-5* mutant worms (Fig. 3F). The splicing of targets specific for SL1 *trans*-splicing (*rps-22, rpl-22, rps-3 and egal-1*) and SL2 *trans*-splicing (*rla-1*) was monitored using RT-PCR (*trans*-splicing of *rps-3* and *rla-1* was reported previously [26]). We also attempted to detect non-*trans*-spliced transcripts of *rps-22* and *rpl-22*; interestingly, an accumulation of non-*trans*-spliced *rps-22* pre-mRNA was seen in the *ubl5* mutants (Fig. 3E). In general, detecting non-*trans*-spliced pre-mRNA is quite challenging, presumably due to rapid processing or instability. Furthermore, the amount of *trans*-spliced *rps-3* and *egal-1* transcripts was comparatively lower in the *ubl5* mutant than in WT animals (Fig. 3G). Nevertheless, the splicing defect in the *ubl5* mutant was neither complete nor seen for every transcript. These data suggest some specificity in the UBL-5 targets in *C. elegans* and its regulatory activity in pre-mRNA splicing. The pre-dominant type of *trans*-splicing defect is unclear due to the limited number of targets analysed. Moreover, from the targets analysed, we did not observe any specific bias towards a particular kind of splicing defects in *ubl-5* mutant worms. Altogether, these data showed pre-mRNA splicing defects in *ubl-5* mutant animals, suggesting that UBL-5 is required for selected *cis*-, *trans*- and alternative splicing in *C. elegans*.

### *C. elegans* UBL-5 complemented the splicing and growth defects of *S. pombe hub1-1* mutants

**Fig. 4:**
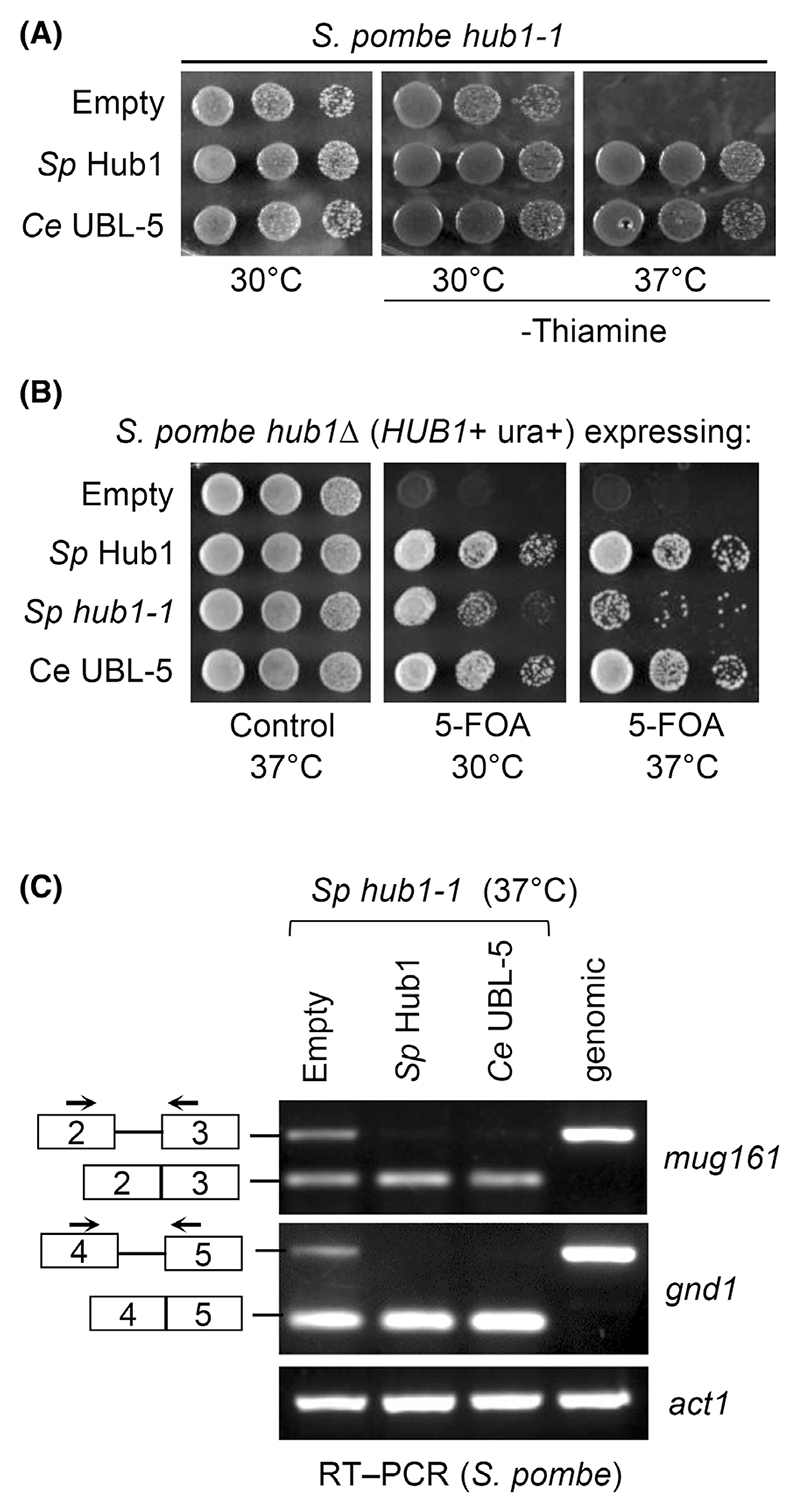
*Caenorhabditis elegans* UBL-5 complements growth and splicing defects in *Schizosaccharomyces pombe hub1 mutant*. (A) Rescue of temperature sensitivity in *S. pombe hub1-1* cells by *C. elegans* UBL-5. A construct expressing *S. pombe hub1* was used as the positive control. The proteins were expressed from the weak *nmt81* promoter. Five-fold serial diluted cells were spotted on the indicated media, followed by incubation at 30 and 37 °C for 4 days. (B) *C. elegans* UBL-5 complements *S. pombe hub1Δ* lethality. Lethality in *S. pombe hub1Δ* cells was rescued by expressing *S. pombe* Hub1 and *C. elegans* UBL-5 at 30 and 37 °C. Five-fold serial diluted cells were spotted on control or FOA-containing plates followed by incubation at 30 and 37 °C for 4 days. Plasmids were expressed from the weak *nmt81* promoter. (C) Rescue of splicing defects in *S. pombe hub1-1* cells by *C. elegans* UBL-5. *S. pombe hub1-1* mutant showed accumulation of intron-containing transcripts for *mug161 and gnd1*. UBL-5 complemented the splicing defect to an extent similar to *S. pombe* Hub1. Block diagrams represent exons and introns. Primers are depicted as arrows. The PCR bands with genomic DNA template show the size expected from pre-mRNAs.

We next studied the functional conservation of *C. elegan*s UBL-5 by complementing Hub1 functions in the fission yeast *S. pombe* (Hub1 is essential for viability and pre-mRNA splicing in *S. pombe*). For this, we expressed *C. elegans* UBL-5 in *hub1* temperature-sensitive and deletion mutants of *S. pombe*. The expression of *C. elegans* UBL-5 rescued the lethality in *S. pombe hub1-1* mutants at 37 °C, similar to the complementation by *S. pombe* Hub1 (Fig. 4A). Interestingly, *C. elegans* UBL-5 also complemented the lethality of *S. pombe hub1Δ* mutant at all temperatures (Fig. 4B). These results indicate that *C. elegans* UBL-5 and *S. pombe* Hub1 are functionally conserved. We also monitored the complementation of the splicing defects in *S. pombe hub1* mutants by the *C. elegans* homologue. RT-PCR-based splicing assays showed restoration of splicing defects in *S. pombe hub1* mutants by the *C. elegans* UBL-5 protein (Fig. 4C). This ability of *C. elegans* UBL-5 to functionally complement the growth and splicing defects of *S. pombe hub1* mutants implies that the protein’s splicing function might be conserved across the eukaryotic kingdom.

## Discussion

### Conserved splicing function of *C. elegans* UBL-5

Through multiple lines of evidence, we have shown that UBL-5 performs a conserved function of RNA splicing in *C. elegans*. Similar to yeast and human cultured cells, the non-covalent interactions of Hub1 with HIND-containing splicing factors are also seen with *C. elegans* proteins. In the budding yeast *S. cerevisiae*, Hub1 is not essential for viability, possibly because of fewer Hub1-dependent introns [16]. In contrast, the protein becomes essential in *S. pombe* and human cells because of a larger number of Hub1-dependent introns [12,16,17]. A similar proposition for UBL-5′s involvement in a larger number of pre-mRNA splicing events can also be suggested for the nematode *C. elegans*, where UBL-5 is also essential for development. Nevertheless, the protein is not critical for all splicing reactions in *C. elegans*. These observations are consistent with the Hub1/UBL5 function in selected pre-mRNAs splicing in yeast and mammalian cells [14,16,17]. Introns containing non-canonical 5’SS in yeast require Hub1 for splicing [16]. Similar features in *C. elegans* and mammalian targets would be interesting to identify. Interestingly, *C. elegans* UBL-5 complemented the lack of Hub1 in *S. pombe* and rescued the growth and splicing defects of *S. pombe hub1* mutants. These observations suggest a conserved splicing function of UBL-5 across eukaryotes.

Splicing defects were missed in *ubl-5* knockdown worms in a previous report [21], possibly because those assays missed analysing the right targets. Alternatively, low levels of UBL-5 protein in the knockdown worms used in the study might have been enough for most of its splicing function. Transcripts containing introns, outron and intercistronic regions have generally been challenging to capture, possibly due to their rapid clearance from the worms. We could partially circumvent these problems by using *ubl-5* knockout worms at the L3 stage and testing against a larger set of genes.

### UBL-5 associates with spliceosomal components

The Hub1–Snu66 interaction is well-studied in *S. cerevisiae* and human cells [14,16]. Besides the interaction with Snu66, Hub1 makes additional contact with Prp5, a DEAD-box helicase, to promote alternative splicing in *S. cerevisiae* [15,18]. *Caenorhabditis elegans* UBL-5 binds both HIND-containing spliceosomal proteins SART-1 and PRP-38. The mode of this interaction through salt bridges also appears to be conserved.

UBL-5 appears to associate with other splicing factors, notably the arginine/serine-rich family proteins, including RSP-1, RSP-2, RSP-3 and RSP-6. It also associates with other splicing factors such as HRPF-1 (hnRNP F homologue) and HRPA-2 (hnRNP A1 homologue). The relevance and mode of these interactions would be interesting to study. We could not detect SART-1 and PRP-38 proteins in the Co-IP experiments. Possible reasons for missing these proteins include their weaker affinities with UBL-5, lower expression levels and technical challenges associated with biochemical purifications from *C. elegans*.

*Trans*-acting splicing factors such as SR proteins bind with the regulatory sequences on pre-mRNA for splicing control [27,28]. In contrast, heterogeneous ribonuclear proteins (hnRNPs) bind to regulatory sequences on exons and inhibit splicing by preventing SR proteins from binding to exons [29,30]. UBL-5 interestingly appears to associate with both types of *trans*-acting splicing factors. Further studies are needed to understand the functional relevance of these interactions.

Human Hub1 has been reported to interact with coilin (a core component of Cajal bodies) and colocalize with Cajal bodies, a subnuclear domain where assembly or modification of spliceosomal components occurs [31]. Therefore, Hub1 might have evolved to perform a broader function in RNA splicing by binding to other spliceosomal proteins in a HIND-independent manner, for example, Prp5 and the SR protein kinases Cdc2/Cdc28-like kinases [32]. Hub1 also associates with proteins through hydrophobic interactions and might function beyond RNA splicing [33].

### Potential role of *C. elegans* UBL-5 in *trans*-splicing

In *C. elegans*, the 5′ ends of many pre-mRNAs undergo *trans*-splicing with either SL1 or SL2 splice leader RNAs, and ~ 85% of the genes undergo *trans*-splicing [7]. Data from our splicing-sensitive microarray suggest a potential role of UBL-5 in *trans*-splicing. SR proteins’ function in *trans*-splicing has been established by *in vitro* splicing assays. They can recruit U2 snRNP to the branch point of natural *trans*-splicing substrates [34,35]. SL snRNPs provide the 5′ splice site, and the pre-mRNA provides the *trans*-splice site (3′ splice site). SL snRNP, U2 snRNP and U4/U6.U5 tri-snRNP assemble into a *trans*-spliceosome to perform *trans*-splicing. Extending the observation from *S. cerevisiae*, where Hub1 interacts with the U1/U2 snRNP factor Prp5 and the tri-snRNP factors Snu66 and Prp38, we hypothesize that UBL-5 might bridge the snRNPs during *trans*-splicing. Moreover, SR proteins also promote the entry of the U4/U6.U5 snRNP into the *cis*-spliceosome [36] and are also critical for the formation of catalytically active *trans*-spliceosome for *trans*-splicing [37]. UBL-5 complexes with SR proteins (RSP-1, RSP-2, RSP-3 and RSP-6); these proteins might facilitate *trans*-spliceosomes assembly for *trans*-splicing selected transcripts in *C. elegans*.

In conclusion, in most eukaryotes, Hub1-binding sites, HINDs, are located in the homologues of the RNA splicing factor Snu66/SART1. However, the plant Snu66 homologue lacks HIND, but this absence is likely compensated by the HIND in the splicing factor Prp38 [16]. Interestingly, in organisms such as *Plasmodium* and *C. elegans*, HINDs are present in the homologues of both Snu66 and Prp38. The implications of more than one splicing factor associating with Hub1 are not yet clear, but it is tempting to suggest that the dual occurrence of HINDs might be linked to the higher prevalence of *cis*- and *trans*-splicing in these organisms [9]. Supporting this, *C. elegans* UBL-5 appears to play a role in *cis*- and *trans*-splicing.

### Accession codes

Splicing-sensitive microarray data are deposited with GEO (accession number GSE157943). Mass-spectrometry data are deposited with the PRIDE data-base (accession number PXD033914).

## Supplementary Material

Supplementary material

Table S2

## Abbreviations

AS: alternative splicing
BPS: branch-point sequence
CBs: Cajal bodies
Co-IP: co-immunoprecipitation
HIND: Hub1 interaction domain
hnRNP: heterogeneous ribonuclear protein
SL: splice leader
snRNAs: small nuclear RNAs
SR: ser/arg proteins
SS: splice-site
UPRmt: mitochondrial unfolded protein response.
