# Supplementary material for: The ubiquitin-like protein Hub1/UBL-5 functions in pre-mRNA splicing in Caenorhabditis elegans
Source: FEBS Lett. Author manuscript; Available in PMC 2024 Mar 26. (PMC7615767; doi:10.1002/1873-3468.14555)
Supplement: Supplementary material [file EMS194845-supplement-Supplementary_material.docx]

**Supplementary Material**

**The ubiquitin-like protein Hub1/UBL-5 functions in pre-mRNA splicing in *Caenorhabditis elegans***

Kiran Kumar Kolathur, Pallavi Sharma, Nagesh Y. Kadam, Navneet Shahi, Ane Nishitha, Kavita Babu, and Shravan Kumar Mishra

*Hs* Snu66-HIND

*Sc* Snu66-HIND-I

*Sc* Snu66-HIND-II

*Sp* Snu66-HIND

*Ce* SART-1-HIND

*Ce* PRP-38-HIND


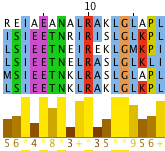


301

318

11

28

7

24

38

55

53

118

70

135

**Supplementary Fig S1**. **Snu66-HIND protein sequence alignment across the organisms.** Clustal Omega multiple sequence alignment of Snu66-HINDs from different organisms. Numbers indicate the positions of amino acids. *Hs, Homo sapiens; Ce, Caenorhabditis elegans; Sp, Schizosaccharomyces pombe; Sc, Saccharomyces cerevisiae*. The alignment visualized with Jalview [39].


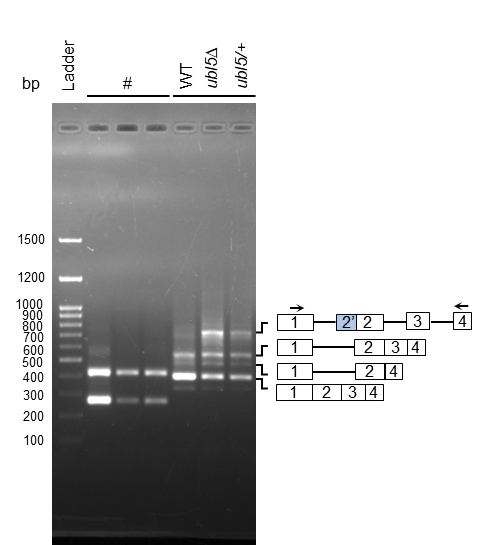


RT–PCR

Pre mRNA 751 bp

Isoform 1 559 bp

Isoform 2 492 bp

Isoform 4 410 bp

*tos-1*

**Supplementary Fig S2**. **Semi-quantitative RT-PCR reveals the accumulation of intron-containing *tos-1* transcripts in *ubl-5* mutants**. The experiment is similar to Fig. 3f. The uncropped gel image of Fig. 3f is shown. #, Unrelated samples.

**Supplementary Table S1**. ***C. elegans* UBL-5 interacts with splicing factors.** The sum of intensity of total peptides for the identified proteins in mass spectrometry. 3xFlag-UBL-5 expressing *C. elegans* cells were immunoprecipitated using anti-FLAG antibody, Co-IP proteins were subjected and analyzed by mass spectrometry. *Ce*, *Caenorhabditis elegans; Hs, Homo sapiens*; *Sc, Saccharomyces- cerevisiae*. – indicates absence of detection.


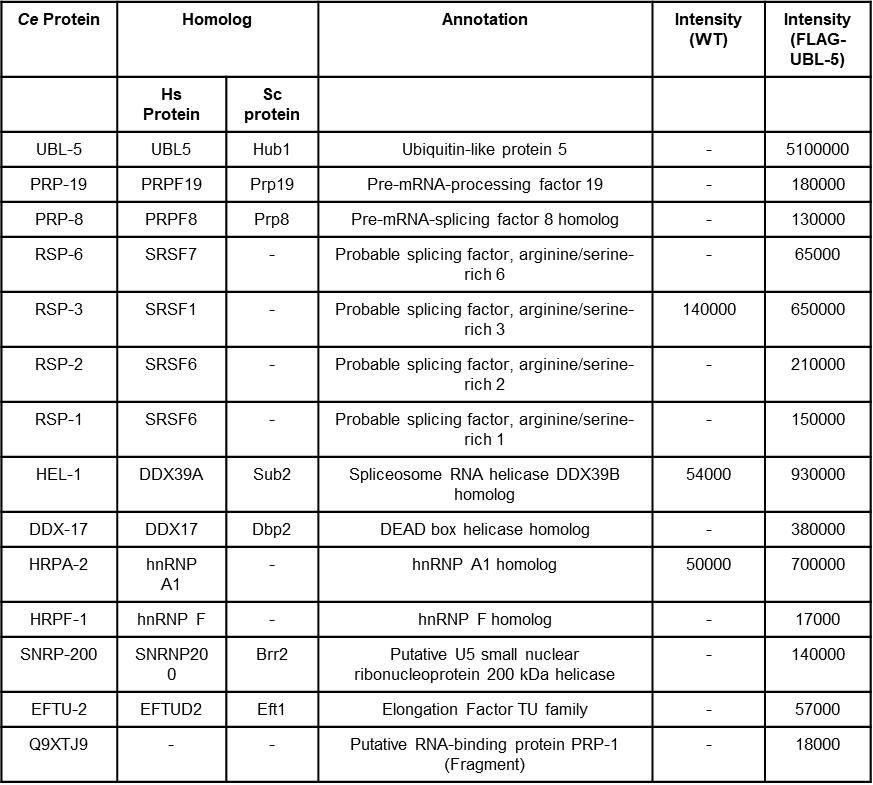


**Supplementary Methods**

**Yeast two-hybrid assay**

All the constructs used in the yeast two-hybrid interaction assays are listed (Table S5). UBL-5 was fused to the DNA binding domain of the pGBDUC1 vector that carries the URA3 selection marker. The HIND domain of SART-1 was cloned with the activation domain of the Gal4p containing vector pGADC1 having a LEU2 selection marker. These constructs were co-transformed (PJ69-7a strain was used) and plated on SC-Leu-Ura plates and incubated for 2-3 days at 30°C. The selected transformants were five-fold serial diluted and spotted on SC-Leu-Ura and SC-Leu-Ura-His plates.

**GST pull-down assay**

In case of *C. elegans* UBL-5-SART-1 interaction, bacterial purified recombinant GST and GST- SART-1(HIND) proteins were individually mixed with 6xHis-UBL-5 protein. The mixture was incubated for 2 hr at 4°C on slow speed rotator. Around 50 µl of pre-washed glutathione (GSH) agarose beads were added to each tube separately and incubated for 1 hr at 4°C on slow speed rotator. After binding with beads, unbound soluble fraction was washed away by centrifugation at 3000 rpm, 4°C for 2-3 min. Later, beads were washed three times with wash buffer 1 (1X PBS with 15% glycerol, 0.5% triton X-100) and finally with wash buffer 2 (without triton X-100). The supernatant was discarded thoroughly using a vaccusip, both inputs (~10%), as well as pull-down proteins, were extracted by heating at 65°C for 10 min in the presence of 30 μl HU buffer (8M urea, 5% SDS, 200 mM Tris pH 6.8, 1mM EDTA, bromophenol blue, and 1.5% DTT). After centrifuging it at 14,000 rpm, 5 min, room temperature; 20 μl of eluted proteins were loaded on NU-PAGE, and pull-down proteins were analyzed by staining the NU-PAGE gel.

**GST pull-down assay using bacterial lysate**

*E. coli* BL21 (DE3) cells were transformed with GST, GST-PRP-38 (HIND), and 6xHis-UBL-5 expressing clones. The selected transformants were cultured at 37°C overnight. These cultures were used as an inoculant for 10 ml of secondary culture starting at OD600 _nm_ of 0.2. *E. coli* cells reached OD 600_nm_ at 0.6-0.8 and were induced with 1 mM IPTG overnight at 16°C. The cells pelleted at 6000xg, 10 min at 4°C were resuspended in 1 ml of B-PER reagent (with 1 mM PMSF, protease inhibitor cocktail solution). Cell pellets were lysed for 10 minutes at RT with intermittent shacking. Lysates were centrifuged at 5000 rpm, 10 min at 4°C, and collected supernatant. The supernatants of GST, 6xHis- UBL-5 and GST-PRP-38 (HIND), 6xHis-Ce UBL-5 were mixed separately for 1 hr at 4°C. 1/10th of the sample mixture was used as input control. 100 µl of pre-washed GSH-beads were added to tubes and mix for 1 hr at 4°C with continuous shaking. Further samples were centrifuge at 3000 rpm for 3 min at 4°C, collected beads were washed 2 times with 1XPBS + glycerol (with 1 mM PMSF), 1 time with 1XPBS + 0.1% triton-X + glycerol (with 1 mM PMSF), and 1 time with 1XPBS + glycerol (with 1 mM PMSF). Finally, the supernatant was discarded thoroughly using vaccusip, pull-down proteins were extracted by heating at 65°C for 10 min in the presence of 50 μl HU buffer. After centrifuging it at 14,000 rpm, 5 min at room temperature; 20 μl of eluted proteins were loaded on NU-PAGE, and pull-down proteins were analyzed by staining the NU-PAGE gel.

**Complementation experiments in *S. pombe***

Preparation of *S. pombe* competent cells, transformation, was performed according to the published protocols for *S. cerevisiae* [40,41]. For the complementation experiment, *S. pombe* cells were transformed with expression constructs containing weak thiamine-repressible promoter *nmt81,* and selected transformants were five-fold serial diluted and spotted on selective agar plates until growth was observed. The absence of thiamine induces the promoter and 5μg/ml of thiamine was used to repress the promoter. In the case of *S. pombe hub1Δ* cells, a *URA4*-bearing plasmid expressing WT *S. pombe* Hub1 was shuffled-out by counter-selection with 5-fluoroorotic acid (FOA; Zymo Research) (1.0 g/L of media) was used in agar plates. Plasmids were expressed from the weak *nmt81* promoter [42].

**Expression and purification of recombinant proteins in *E. coli***

*E. coli* BL21(DE3) cell pre-cultures were inoculated in 50 ml antibiotic-containing LB media and cultured at 37^o^C overnight. These cultures were used as an inoculant for larger volumes starting at OD600_nm_ of 0.2. *E. coli* cells reached OD600_nm_ at 0.4-0.5 were induced with 1 mM IPTG for 3-5 hrs at room temperature.

For GST-fusion proteins, the cells pelleted at 6000xg, 10 min, at 4^o^C were resuspended in 40 ml of lysis buffer (with 1 mM PMSF, 200 µl protease inhibitor cocktail solution, 0.1 µl/ml nuclease) and treated with lysozyme on ice for 30 min. Followed by sonication (10-15 amplitude, 10 second on sonication &10 second on cooling), lysates were centrifuged at 5000 rpm for 10 min at 4^o^C. The supernatants were loaded onto Glutathione Sepharose beads (Qiagen) and incubated for 60 min on ice with continuous shaking. It is transferred to the column (10 ml capacity) and subsequently, columns were washed 3-4 times with 10 ml of lysis buffer, 2 times with 10 ml of high salt buffer (with 300 mM NaCl), once with 10 ml of detergent buffer (with 1% triton X-100) and finally with 10 ml of PBS. Finally, proteins were eluted with 500 µl of GST elution buffer. Total 6 such fractions were eluted; protein concentration was measured and SDS PAGE was performed to analyze the purity of the purification. The eluted was dialyzed against PBS (0.1 mM DTT + 0.2 mM PMSF) for 2-3 hrs at 4^o^C and later shifted to glycerol-containing PBS buffer for 16 hrs (0.1 mM DTT + 0.2 mM PMSF + 10% glycerol) prior to freezing in liquid N_2_ and storage at -80^o^ C.

For purification of insoluble 6xHis-tagged fusion proteins, denaturing conditions were applied for its purification. *E. coli* cells were harvested (as above), cell pellets were dissolved in Ni-NTA lysis buffer, and lysozyme digestion and sonication were performed (as above). After centrifugation, pellet fractions were washed with 1xPBS and resuspended in high urea buffer (8M urea + 1XPBS) for 2-3 hrs at 37^o^C. Cleared lysates after centrifugation were incubated with Ni-NTA agarose beads (Qiagen) for 1 h at 4^o^C. Followed by 4-5 times washing with 10 ml of wash buffer (20 mM imidazole, 8M urea in 1xPBS), Finally, affinity matrix-bound proteins were eluted with 500 µl of Ni-NTA elution buffer with 8M urea. Total 6 such fractions were eluted. The eluted was dialyzed against 1x PBS with 4M urea for 1 hr at 4^o^C, shifted to 1x PBS with 2M urea for 1 hr at 4^o^C and later shifted to glycerol-containing PBS buffer for 16 hrs (0.1 mM DTT + 0.2 mM PMSF + 10% glycerol). Dialysed samples were analyzed and stored as above.

**RNA isolation and RT-PCR**

RNA isolation and cDNA synthesis was carried out as described earlier [43]. Briefly, cells were grown at 30°C until OD600_nm_ around 5 (37°C temperature shift for 15 minutes was carried out). Yeast cells were harvested by a rapid filtration process, total RNA extraction was carried by hot acid phenol method and clean-up of RNA was carried out by the Zymo-Spin II column (Zymo Research). 3µg total RNA converted to cDNA using random hexamer primers (Invitrogen) and reverse transcriptase (RT) at 42°C for 16 hours. Using specific primers listed (Table. S6), RT-PCR assays were carried out, and the analysis of PCR and the products by agarose gel electrophoresis.

### *C. elegans* strains

Strains were maintained at 20^o^C, as described previously [44]. The strains used in this study are the Bristol N2 strain that was used as the WT control strain and the *ubl-5(gk3358*) was used as the mutant strain (VC3368 from CGC, 3X outcrossed).

The *ubl-5 (gk3358)* allele is a homozygous lethal deletion and is balanced by bli-4- and GFP-marked translocation. Heterozygotes are WT with pharyngeal GFP signal and segregate WT GFP, arrested hT2 aneuploids, and non-GFP *gk3358* homozygotes (early larval arrest). Homozygous hT2[bli-4 let-? qIs48] is inviable.

### Co-immunoprecipitation (Co-IP) assays using worm lysates

Worms from plates were washed thrice with M9 buffer and transferred to a 1.5 ml microcentrifuge tube, the supernatant was discarded, and cell pellets were dissolved in 0.4 ml *C. elegans* HEPES lysis buffer per plate of worms (PMSF, protease inhibitor, phosphatase inhibitor). Further froze in liquid nitrogen and stored -80^o^C and lysates were transferred to steel dounce homogenizer on ice, stroked 30-40 times. Further lysates were subjected to homogenization using mortar and pestle. Around 0.2 ml of lysis buffer (volume of 1.5X lysis buffer with protease inhibitors) to each gram of adult/embryo. The worm lysate was centrifuged for 10 minutes at 5000 rpm at 4^o^C. Protein concentration was estimated using bradford reagent. The experiment was as described previously [16,45]. (Note: HEPES buffer with 0.5% triton x-100 was used in washing steps).

Destained gel to a clear background so that bands can be easily seen and excised gel band is placed into a microcentrifuge tube with some sterile double distilled water.

**Methods for Protein Sequence Analysis by LC-MS/MS performed by Harvard Medical School, Taplin Mass Spectrometry Facility, Boston, MA.**

Excised gel bands were cut into approximately 1 mm^3^ pieces. Gel pieces were then subjected to a modified in-gel trypsin digestion procedure [46]. Gel pieces were washed and dehydrated with acetonitrile for 10 min. followed by removal of acetonitrile. Pieces were then completely dried in a speed-vac. Rehydration of the gel pieces was with 50 mM ammonium bicarbonate solution containing 12.5 ng/µl modified sequencing-grade trypsin (Promega, Madison, WI) at 4ºC. After 45 min., the excess trypsin solution was removed and replaced with 50 mM ammonium bicarbonate solution to just cover the gel pieces. Samples were then placed in a 37ºC room overnight. Peptides were later extracted by removing the ammonium bicarbonate solution, followed by one wash with a solution containing 50% acetonitrile and 1% formic acid. The extracts were then dried in a speed-vac (~1 hr). The samples were then stored at 4ºC until analysis.

On the day of analysis, the samples were reconstituted in 5 - 10 µl of HPLC solvent A (2.5% acetonitrile, 0.1% formic acid). A nano-scale reverse-phase HPLC capillary column was created by packing 2.6 µm C18 spherical silica beads into a fused silica capillary (100 µm inner diameter x ~30 cm length) with a flame-drawn tip [47]. After equilibrating the column each sample was loaded via a Famos auto sampler (LC Packings, San Francisco CA) onto the column. A gradient was formed and peptides were eluted with increasing concentrations of solvent B (97.5% acetonitrile, 0.1% formic acid).

As peptides eluted, they were subjected to electrospray ionization and then entered into an LTQ Orbitrap Velos Pro ion-trap mass spectrometer (Thermo Fisher Scientific, Waltham, MA). Peptides were detected, isolated, and fragmented to produce a tandem mass spectrum of specific fragment ions for each peptide. Peptide sequences (and hence protein identity) were determined by matching protein databases with the acquired fragmentation pattern by the software program, Sequest (Thermo Fisher Scientific, Waltham, MA) [48]. The sum intensity is the summation of the peak heights for each peptide matched to a protein. All databases include a reversed version of all the sequences and the data was filtered to between a one and two percent peptide false discovery rate.

Those data (Table S1 and S2) were a qualitative comparison of protein levels between the control and UBL5 co-IP samples based on the number of peptides and the intensity values.  The sum intensity values for each protein are the summation of all the peaks (peak heights or peak intensities) of all the peptides matched to that protein. Based on the total signal, splicing factors enriched in UBL5 IP are shown in Table S1. In Table S2, S14 refers to WT, and S15 refers to Flag-UBL-5 samples. Table S2 represents all the proteins detected by the mass spectrometer.

***C. elegans* RNA isolation**

*C. elegans* were grown on OP50 containing plates for mixed stage populations of worms. The worms were harvested by washing with an M9 buffer in a microcentrifuge tube. Centrifuging at 14,000 rpm pelleted the worms down. The worms were washed 2-3 times to get rid of OP50. For stage, specific RNA isolation worm populations were first synchronized by bleaching the worms, and then the worms are collected at different time points to collect L1, L2, L3, L4, and adult worms. The RNA isolation performed similar to above [43].

**Quantitative PCR**

The total RNA was extracted using Trizol from wild-type worms in a stage-specific manner by Qiagen RNA easy kit as per the manufacturer’s instructions. cDNA synthesis (from 50ng of total RNA) was performed using the Roche Transcriptor high fidelity cDNA synthesis kit (Roche; # 05081955001). qPCR reactions were performed using the SYBR real-time PCR kit. qPCR was done using Roche LightCycler 480. Ct values were calculated using Δ C_t_ = (Ct_GOI_ – Ct_HG_) with the Roche software. Fold expression is considered as 2^-△Ct^. GOI indicates the gene of interest, and HG indicates the housekeeping gene, *act-1*. The transcript level of *ubl-5* was compared using the housekeeping gene, actin as a reference. Real-time experiments were biological replicates performed in triplicate.

**cDNA preparation and RT-PCR Experiment:** cDNA preparation was done using the protocol described by the Verso cDNA synthesis kit (AB-1453/B). For this, an RNA equivalent of 800 ng/µl was reverse transcribed using a combination of random hexamers and oligo(dt) primers. As this kit has an in-built DNase enzyme, inactivation at 95^o^C was done for 2 minutes after incubation at 42^o^C for 30 minutes.

For Reverse Transcription-PCR, RedTaq Jumpstart polymerase (R2523- Sigma Aldrich) was used to amplify the cDNA (800ng per 10µl reaction). Gene-specific primers for the targets were used with the housekeeping gene control (Table. S6).

***ubl-5* rescue experiments**

The *ubl-5* genomic DNA was cloned into the pPD49.26 vector backbone. The *ubl-5* mutant line was used for transforming the *ubl-5* genomic DNA construct. The Transformations were performed using microinjections as described previously [49]. The rescue constructs were injected in concentrations of 10–20 ng/μl. p*myo-2*::mCherry (2 ng/μl) was used as a co injection marker. The transformed animals were then grown for 3 generations on OP50 bacteria-containing plates. For performing the rescue experiment single adult *C. elegans* were transferred to a fresh OP50 containing plate and allowed to lay progeny. After 24 hours, the worm was transferred to a fresh plate and the number of live progeny present on the previous plate was counted. A similar process was repeated for 3 days. Similarly, we counted the number of progeny for WT, *ubl-5/+,* and *Δubl-5* worms.

The strain used for rescue experiments is the *ubl-5 (gk3358)* worms. The *ubl-5 (gk3358)* worms lay progeny with the *ubl-5/+* and *ubl-5* genotypes. We can easily identify the two genotypes with the help of fluorescent markers. After injecting the *ubl-5 (gk3358)* worms with the rescue plasmid, we looked for the *ubl-5* mutant worms which are viable even after the L3 stage and counted them.

This strain was provided by the *C. elegans* Reverse Genetics Core Facility at the University of British Columbia, which is part of the international *C. elegans* Gene Knockout Consortium.

### *C. elegans* splicing-sensitive microarray

The microarray experiment was carried out at the microarray facility of genotypic technology, Bengaluru, India (Agilent certified). RNA isolation was done as described previously [43]. Double-stranded cDNA was synthesized from 500ng each of total RNA using oligo dT primer tagged to a T7 polymerase promoter and reverse transcriptase at 40°C. Using in vitro transcription, cRNA was synthesized from double-stranded cDNA, during this process the dye Cy3 CTP (Agilent) was incorporated using Quick-Amp labeling Kit (Agilent, p/n5190-0442). Labeled cRNA was cleaned up by Qiagen RNeasy columns (Qiagen, Cat No: 74106) and quality was evaluated using the Nanodrop ND-2000. Fragmentation of labeled cRNA and hybridization was performed using the gene expression hybridization kit (In situ Hybridization kit, Agilent, Part Number 5190-0404). Hybridization onto an Agilent gene expression Microarray 8X60K was carried out in Agilent’s surehyb chambers at 65°C for 16 hours. The hybridized slides were washed using gene expression wash buffers and scanned using the microarray scanner (Agilent Technologies, Part Number G2600D). The array has been designed to study pre-mRNA splicing of Caenorhabditis elegans using 6,280 transcripts from the wormbase database. The array format is 8x60K, which comprises a total number of 62,976 probes, including replicated probes along with 1319 Agilent control probes. The array consists of exon, exon-ligated, intron, *trans*-splicing, and splice variant probes. Three replicates for WT and mutant samples were analyzed.

Outrons are indeed the 5’ untranslated regions that end in SL1 *trans*-splice sites and should be explicitly used in this context. SL2 *trans*-splicing, is reserved for splicing downstream genes in polycistronic pre-mRNAs derived from cotranscribed gene clusters or operons. For looking at the SL2 *trans*-splicing, we have used a 60 bp sequence, 30 bp upstream and 30 bp downstream of the *trans* splice site of the candidate gene as a probe.

The source of the sequence is from the wormbase. Along with the Genotypic Technology Pvt. Ltd. Bangalore, we have custom designed our own microarray, and the Agilent Technologies International Pvt Ltd, India, pinned microarray probes.

**Microarray Data Analysis**

Feature extracted raw data was examined using Agilent genespring GX software. Data normalization was carried out in genespring GX using the 75th percentile shift method and fold change values were acquired by analyzing the ratio of mutant samples with respect to specific wild-type samples. Significant genes up-regulated fold> 1 (logbase2) and down-regulated <-1 (logbase2) were determined. Statistical student T-test p-value among the replicates was calculated based on the volcano Plot Algorithm. Heatmaps were generated considering entity-wise clustering by using Agilent genespring GX software.

Normalization of the data was done in GeneSpring GX using the 75th percentile shift method for the individual array. After 75tth percentile normalization, the normalized values are used for fold calculation of Fold expression values. We have experimented with replicates for WT and mutant samples. The fold expression values for WT and mutant were obtained as follows: 1. The median of 75th normalized expression values of WT replicate samples is calculated for each probe. The 75th normalized expression value of each probe in the individual WT sample is subtracted by the median calculated value of WT samples to obtain the fold expression value. We got the individual expression values for all three WT samples for all the probes in the array, and the geometric mean of fold values is calculated for all the WT replicates. 2. The 75th normalized expression value of each probe in the individual mutant sample is subtracted by the median calculated value of WT samples to obtain the fold expression value. We obtained the individual expression values for all mutant samples for all the probes in the array, and the geometric mean of fold values is calculated for all the mutant replicates. The complete data is included (Table. S10).

**Supplementary Table S3 *S. pombe* strains used in this study**

| **Strain** | **Relevant genotype** | **Reference** |
| --- | --- | --- |
| SP10 | PEM2 *hub1-I42S::Nat-NT2* | [45] |
| SP13 | *JY741 Δhub1::aur1R pUR19-hub1+* | [12] |

**Supplementary Table S4 *S. cerevisiae* strain used in this study**

| **Strain** | **Relevant genotype** | **Reference** |
| --- | --- | --- |
| PJ69-7A | *trp1-901 leu2-3,112 ura3-53 his3-200 gal4 gal80 GAL1::HIS3 GAL2- ADE2 met2::GAL7-lacZ* | [50] |

**Supplementary Table S5 Plasmid clones used in this study**

| **Plasmid No** | **Name** | **Description** | **Reference** |
| --- | --- | --- | --- |
| D121 | pREP81x*-Ce ubl-5* | *C. elegans ubl-5* in pREP81X | This study |
| D122 | pREP81x-*Sp hub1* | *S. pombe hub1* in pREP81x | This study |
| D123 | pGBDUC1-*Ce ubl-5* | *C. elegans* *ubl-5* in pGBUC1 | This study |
| D127 | pET28a-*6xHIS-Ce ubl-5* | *C. elegans* *ubl-5* in pET28a | This study |
| D150 | pGEX-5x-1*-* SART-1(HIND) | *C. elegans* HIND (aa 1-80) in pGEX-5x-1 | This study |
| D151 | pGADC1-*Ce sart-1(HIND)* | *C. elegans* HIND (aa 1-80) in pGADC1 | This study |
| D163 | pGBDUC1*-Ce* UBL-5(D22A) | *C. elegans* UBL-5(D22A) in pGDAC1 | This study |
| D169 | pGEX-5x-1*-Ce prp38(HIND)* | *C. elegans* HIND (aa 291-320*)* in pGEX-5x-1 | This study |
| D172 | pGADC1-*Ce* SART-1 (HINDR62A) | *C. elegans* HIND-R62A *in* pGADC1 | This study |
| D174 | pREP81x*-hub1I42S* | *S. pombe hub1-I42S* in pREP81x | This study |
| D280 | pPD49.26-3XFLAG*-Ce ubl-5* | *C. elegans ubl-5 in pPD49.26* | This study |

**Supplementary Table S6. List of primers used in this study (F-forward primer; R-reverse primer) (Int-intron; Ex-exon)**

| **Number** | **Name** | **Sequence (5’-3’)** |
| --- | --- | --- |
| SKM_PR 13 | *act1* F | CCCCTAGAGCTGTATTCCC |
| SKM_PR 14 | *act1* R | CAGTGGTACGACCAGAGG |
| SKM_PR 1235 | *gnd1 exon 4 F* | CCGTACAACTTCCAGAGTTGACGAG |
| SKM_PR 1236 | *gnd1 exon 5 R* | CAAATTCCTCAAGGGAGTGAGCACC |
| SKM_PR 1463 | *mug161 Ex2 F* | CAAGCGTACAACTAGTGCGG |
| SKM_PR 1464 | *mug161 Ex3 R* | AATGGACTCTGGCAAACCAGC |
| Tos-1-F | *tos-1 F* | ATGATCTACGGATTCGAGTCGTCACCATC |
| Tos-1-R | *tos-1 R* | GAAGAAATCTTCCAGTCCGAAGGG |
| B0350.2b-F | *b0350.2b F* | TTCCAGAAGACGTCGAGCAAA |
| B0350.2b-R | *b0350.2b R* | ACCTTCGTGATCTTCATGTTG |
| SL1-F | *SL1-F* | GGTTTAATTACCCAAGTTTGAG |
| SL2-F | *SL2-F* | GGTTTTAACCCAGTTACTCAAG |
| Rps-3-R | *rps-3-R* | CTCTTTTGGACAACCGAGGTGAGC |
| Rla-1-R | *rla-1-R* | GGAAGAGACAGAAGTGATGAGG |
| Egal-1-R | *egal-1-R* | CTGCAAAAACTCGGTTAATC |
| Rpl-22 | *rpl-22-F (outron)* | AAATGAGTAGAGTTCTCGGC |
| Rpl-22 | *rpl-22-R* | AAGATCTTCGATTCTGAGAATTCC |
| Rps-22 | *rps-22-F (outron)* | GAAGTTTTTCACATACTTTTCTCG |
| Rps-22 | *rps-22-R* | GGACGGATGAGAACCTGAC |
| Gpd-2 | *gpd-2-F* | CTCCATCGACTACATGGTCTACTTG |
| Gpd-2 | *gpd-2-R* | AGCTGGGTCTCTTGAGTTGTAGAC |

**Supplementary Table S7 List of primers used in this study specifically for *C. elegans* (F-forward primer; R-reverse primer) (Int-intron; Ex-exon)**

| BTL9 | UBL-5 | CGCGGATCCATGATTGAAATCACA  GTAAACG | Cloning BamH1-F | pET28a |
| --- | --- | --- | --- | --- |
| BTL10 | UBL-5 | GCGTCGACTCATTGGTAGTAGAGCTCG | Cloning  SalI-R | pET28a |
| BTL11 | SART-1 | CGCGGATCCATGTCTTCCAAATACTC | Cloning BamH1-F | pGEX-5x |
| BTL12 | SART-1 | GCGTCGACTCAAGCATCACGTTCC | Cloning  SalI-R | pGEX-5x |
| BTL13 | UBL-5 | CGGGATCCATGATTGAAATCACAGTAAACG | Cloning  BamH1-F | pGADC1 |
| BTL14 | UBL-5 | AACTGCAGTCATTGGTAGTAGAGCTCG | Cloning  PstI-R | pGADC1 |
| BTL15 | SART-1 | CGGGATCCATGTCTTCCAAATACTCA | Cloning  BamH1-F | pGBDUC1 |
| BTL16 | SART-1 | AACTGCAGTCAAGCATCACGTTCC | Cloning  PstI-R | pGBDUC1 |
| BTL31 | *ubl-5* | AATCCCTCGTGAATCTCGTAATCCAT | Genotyping External-F |  |
| BTL32 | *ubl-5* | TTCAAATCTCACCGTGTTCCAG | Genotyping Internal-F |  |
| BTL33 | *ubl-5* | GCAAGTGTGAAACGCTATGTTC | Genotyping External-R |  |
| PS305 | *ubl-5* | ATGATTGAAATCACAGTAAACGATCG | qPCR |  |
| PS306 | *ubl-5* | TAGAGCTCGAAATTGAATCCCTCG | qPCR |  |
| PS422 | *ubl-5*  gDNA | AATTAGGTACCTTGGTAGTAGAGCTCG  AAATTGAATC | Cloning  KpnI-R | pPD49.26 |
| PS423 | *ubl-5*  gDNA | AATTTACCTGCAGGGACTCTCCAGAA  GAAACTTCA | Cloning  SbfI-F | pPD49.26 |

# Supplementary References

39. Waterhouse AM, Procter JB, Martin DMA, Clamp M, Barton GJ. Jalview Version 2--a multiple sequence alignment editor and analysis workbench. Bioinformatics. 2009;25:1189–91.

40. Janke C, Magiera MM, Rathfelder N, Taxis C, Reber S, Maekawa H, et al. A versatile toolbox for PCR-based tagging of yeast genes: New fluorescent proteins, more markers and promoter substitution cassettes. Yeast. 2004;21:947–62.

41. Knop M, Siegers K, Pereira G, Zachariae W, Winsor B, Nasmyth K, et al. Epitope tagging of yeast genes using a PCR-based strategy: More tags and improved practical routines. Yeast. 1999;15:963–72.

42. Sajeevan A, Pandian R, Mishra SK. Vectors with a flexible multiple cloning site and modular epitope tags for gene expression studies in Schizosaccharomyces pombe. Gene Reports [Internet]. 2022;29:101681. Available from: https://www.sciencedirect.com/science/article/pii/S2452014422001893

43. Inada M, Pleiss JA. Genome-wide approaches to monitor pre-mrna splicing. 2nd ed. Methods Enzymol. Elsevier Inc.; 2010.

44. Brenner S. The genetics of Caenorhabditis elegans. Genetics. 1974;77:71–94.

45. Thakran P, Pandit PA, Datta S, Kolathur KK, Pleiss JA, Mishra SK. Sde2 is an intron‐specific pre‐ mRNA splicing regulator activated by ubiquitin‐like processing . EMBO J. 2018;37:89–101.

46. Shevchenko A, Wilm M, Vorm O, Mann M. Mass spectrometric sequencing of proteins silver-stained polyacrylamide gels. Anal Chem. United States; 1996;68:850–8.

47. Peng J, Gygi SP. Proteomics: the move to mixtures. J Mass Spectrom. England; 2001;36:1083–91.

48. Eng JK, McCormack AL, Yates JR. An approach to correlate tandem mass spectral data of peptides with amino acid sequences in a protein database. J Am Soc Mass Spectrom. United States; 1994;5:976–89.

49. Mello CC, Kramer JM, Stinchcomb D, Ambros V. Efficient gene transfer in C.elegans: Extrachromosomal maintenance and integration of transforming sequences. EMBO J. 1991;10:3959–70.

50. James P, Halladay J, Craig EA. Genomic Libraries and a Host Strain Designed for Highly Efficient Two-Hybrid Selection in Yeast. Genetics. 1996;144:1425–36.
